# The Effect of In Situ Heat Treatment on the Microstructure and Mechanical Properties of H13 Tool Steel Specimens Produced by Laser-Engineered Net Shaping (LENS^®^)

**DOI:** 10.3390/ma18225164

**Published:** 2025-11-13

**Authors:** Michalina Rothen-Chaja, Izabela Kunce, Agata Radziwonko, Tomasz Płociński, Julita Dworecka-Wójcik, Marek Polański

**Affiliations:** 1 Posalux SA, 18 F. Oppliger Street, P.O. Box 6075, CH-2500 Biel, Switzerland; 2 Corrosion and Chemistry Department, Road and Bridge Research Institute, 1 Instytutowa Street, 03-302 Warsaw, Poland; izabela.kunce@ibdim.edu.pl; 3 Department of Functional Materials and Hydrogen Technology, Military University of Technology, 2 Kaliskiego Street, 00-908 Warsaw, Poland; agata.radziwonko@wat.edu.pl (A.R.); julita.dworecka@wat.edu.pl (J.D.-W.); 4 Faculty of Materials Science and Engineering, Warsaw University of Technology, 141 Wołoska, 02-507 Warsaw, Poland; tomasz.plocinski@pw.edu.pl

**Keywords:** additive manufacturing, H13 tool steel, heat treatment, tempering, martensitic transformation, mechanical properties, direct energy deposition, laser cladding, LENS^®^, microstructure

## Abstract

Samples of H13 tool steel were produced using the LENS^®^ laser additive manufacturing technique. Three variants of samples were produced such that during and 2 h after deposition, both the substrate and sample temperatures were maintained at 80, 180, and 350 °C. After the samples were produced, the effect of the substrate temperature on their metallurgical quality, microstructure, and mechanical properties was determined. No segregation of alloying elements was observed. The test results indicate that, depending on the temperature used, the structure of the H13 alloy is martensitic or martensitic-bainitic with a slight residual austenite content of up to 2.1%. Owing to structural changes, the obtained alloy is characterized by lower impact strength compared with conventionally produced alloys and high brittleness, particularly when using an annealing temperature of 350 °C. Isothermal annealing above the martensite start temperature results in extreme brittleness due to a partial structural transformation of martensite into bainite and probable carbide precipitation processes at the nanoscale.

## 1. Introduction

H13 (X40CrMoV5-1, DIN 1.2344) steel, a chromium–molybdenum hot-work tool steel, is widely recognized for its superior mechanical properties, including exceptional hardness, toughness, and thermal stability. Characterized by its composition—approximately 5% Cr, 1.4% Mo, and 0.4% V—H13 steel is designed to withstand high-temperature applications while maintaining its dimensional stability and wear resistance. These attributes make H13 steel particularly suitable for die casting, extrusion dies, and other tooling applications where high levels of thermal and mechanical stresses are prevalent. The material is characterized by its good balance of hardness and toughness, which are crucial for components subjected to cyclic thermal loads and mechanical stress [1].

Additive manufacturing techniques, including directed energy deposition (DED) and selective laser melting (SLM), are increasingly employed to produce complex geometries from H13 steel. These techniques enable the layer-by-layer fabrication of parts, allowing for design flexibility that traditional manufacturing methods cannot match, as well as several advantages, such as reduced material waste and shorter lead times in production [2]. During the fabrication of steels via additive manufacturing, in addition to structural changes induced by high cooling rates, previously deposited layers are thermally affected by remelting and reheating during the deposition of subsequent layers. These thermal cycles have a significant influence on the resulting microstructure and mechanical properties [3]. For H13 tool steel, as with other alloys produced using additive techniques, the rapid cooling during solidification promotes the formation of a layered dendritic or cellular–dendritic microstructure. However, this process is often accompanied by high levels of internal stress, porosity, and cracking, which represent critical challenges in the additive manufacturing of tool steels [4,5,6]. The predominant phases observed in the microstructure of additively manufactured H13 steel include martensite, retained austenite, and, depending on the specific processing parameters, carbides [7] or nanocarbides [8,9].

To reduce the impact of rapid cooling on the high level of internal stresses in manufactured parts, the substrate or powder bed can be preheated during part manufacturing, depending on the manufacturing method. Martens et al. [10] showed that preheating temperatures up to 200 °C during the SLM process soften the material, whereas when the preheating temperatures increase, compressive residual stresses gradually evolve to a tensile nature. Parts produced using a preheating temperature of 400 °C have a homogeneous bainitic microstructure and ultimate tensile strength (UTS) similar to conventionally produced and tempered alloys but with higher hardness. Up to a preheating temperature of 300 °C, no structural changes are observed in SLM-produced parts [10,11], although some literature reports indicate that a preheating temperature of 200 °C results in an increase in the amount of retained austenite compared with non-preheated samples [12].

A critical heat-treatment technique applied to H13 steel to enhance its mechanical properties is isothermal annealing. This ensures that the microstructure undergoes controlled transformation, resulting in a uniform distribution of tempered martensite, which optimizes the balance between hardness and toughness. The effects of isothermal tempering on H13 steel can vary significantly depending on the tempering temperature and cooling rate [13]. Tempering at temperatures above 550 °C results in the decomposition of retained austenite and secondary carbide precipitation [14], as well as changes in the mechanical properties of H13 steel. For example, Wen et al. [15] observed that samples tempered at 600 °C for 1 h exhibited increased yield strength (1647 MPa) and UTS (2013 MPa) compared with as-SLM-produced H13 steel. However, the elongation-to-break metric decreased from 8.5% to 4.1% because fine Cr23C6 precipitates pinned the boundaries of cellular structures, fine grains, and high-density dislocations. An improvement in elongation of up to 12.3% has been achieved through the decomposition of martensite and the recovery of dislocation density by tempering at 700 °C for 1 h. The low-temperature tempering at 200 °C of H13 tool steel after solution treatment significantly reduces the hardness relative to SLM samples and the growth of M7C3 carbides without any new initiation of precipitates during the tempering [16].

Additively manufactured H13 typically solidifies into a martensitic matrix with retained austenite and carbides, but the steep thermal gradients and cyclic reheating between layers cause high residual stresses, porosity, and cracking; hence, the routine relies on post-build stress relief and tempering. While substrate preheating and in situ thermal management are well established in laser powder bed fusion (LPBF), comparable strategies remain far less explored in DED/LENS^®^ because most systems lack tightly integrated, high-temperature platform control and studies have focused on parameter windows or post-deposition heat treatments. The limited DED literature that incorporates active in situ heating (e.g., heating plates or induction) [17,18] demonstrates clear benefits—lower cooling rates, reduced cracking, and altered phase evolution—but is focused on other tool steels or maraging grades rather than H13 and seldom targets controlled tempering during the build itself [19]. Recent reports on in situ thermal exposure in DED show that tailored thermal histories can precipitate strengthening phases and harden the matrix during fabrication, supporting the premise that “in situ heat treatment” is feasible.

Building on these insights, our study deliberately operated LENS^®^ with a heated substrate to impose a designed in situ heat-treatment profile during the deposition of H13. The objectives were to (i) moderate cooling rates to suppress crack-driving thermal stresses, (ii) promote partial tempering of as-formed martensite and stabilize retained austenite, and (iii) steer carbide/nanocarbide precipitation toward a tougher, more fatigue-resistant microstructure—without a separate furnace step. By converting LENS^®^ from a purely shape-creation process into a coupled “build-and-treat” route, we targeted property parity with conventionally tempered H13 while retaining the geometrical freedom and repair capability of DED. To the best of our knowledge, such tightly controlled, high-temperature substrate heating used to achieve in situ tempering of H13 during DED is rare and represents a significant advancement in thermal management for tool steels in DED.

## 2. Materials and Experimental Procedures

**Figure 1:**
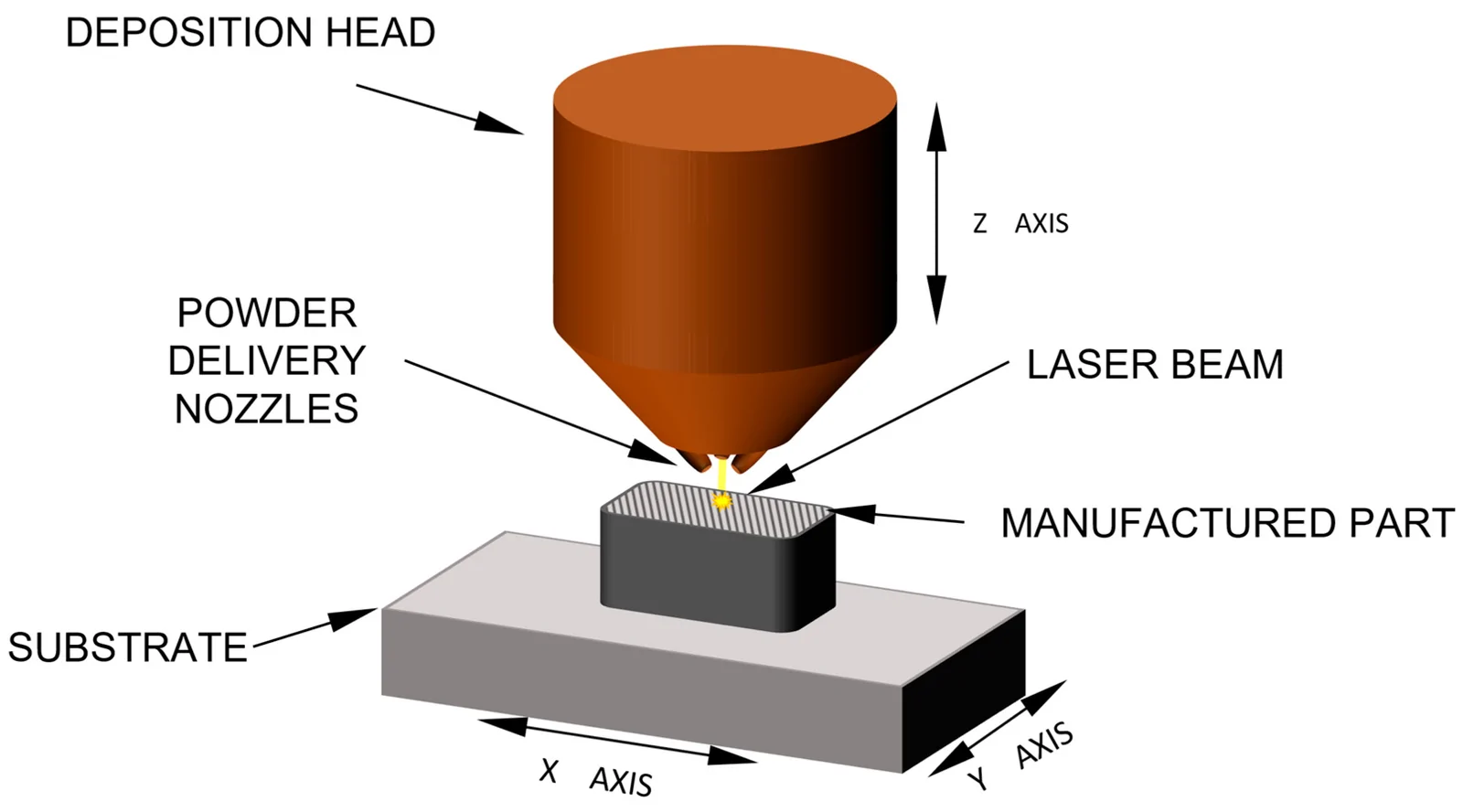
Schematic of the LENS additive manufacturing process. The figure is based on [20].

An MR-7 (Optomec, Albuquerque, NM, USA) equipped with a fiber laser with a maximum power of 500 W was used to prepare the samples using the LENS^®^ additive laser shaping technique. Figure 1 shows a general scheme of the LENS additive manufacturing process [20]. During the production of samples or parts, metal powders are fed into a molten pool, which is formed by a focused laser beam. The samples were built in a protective atmosphere of argon, and the oxygen content in the atmosphere during the building of the samples was kept below 2 ppm. A 15 mm thick flat plate of X37CrMoV5-1 hot-work tool steel with a similar chemical composition to X40CrMoV5-1 steel was used as the substrate. A series of specimens were formed with dimensions of 47 mm × 23 mm × 11 mm (X × Y × Z), and the specimen fabrication parameters used were as follows: power: 320 W, feed/contour head speed: 8 mm/s, powder output: 7.5–8 g/min, hatch space: 0.37 mm, hatch shrink: 0.1 mm, and thickness of the applied layer: 0.25 mm. During the building of the samples, the substrate temperature was maintained at 80, 180, and 350 °C using two heating/cooling tables of our own design. The low-temperature liquid-cooled table enabled temperatures to be maintained from 10 to 85 °C. The high-temperature table, with a heater power of 2000 W, facilitated substrate temperatures in the range of 100–600 °C. Each sample was built in about 4 h to ensure temperature stability during the fabrication process. After each sample was built, the preset substrate temperature was maintained for 2 h to provide conditions for phase transformations to occur in the volume of the entire sample. Owing to the low height of the sample, we assumed that the temperature over its volume remained stable, with the value corresponding to that of the substrate.

**Figure 2:**
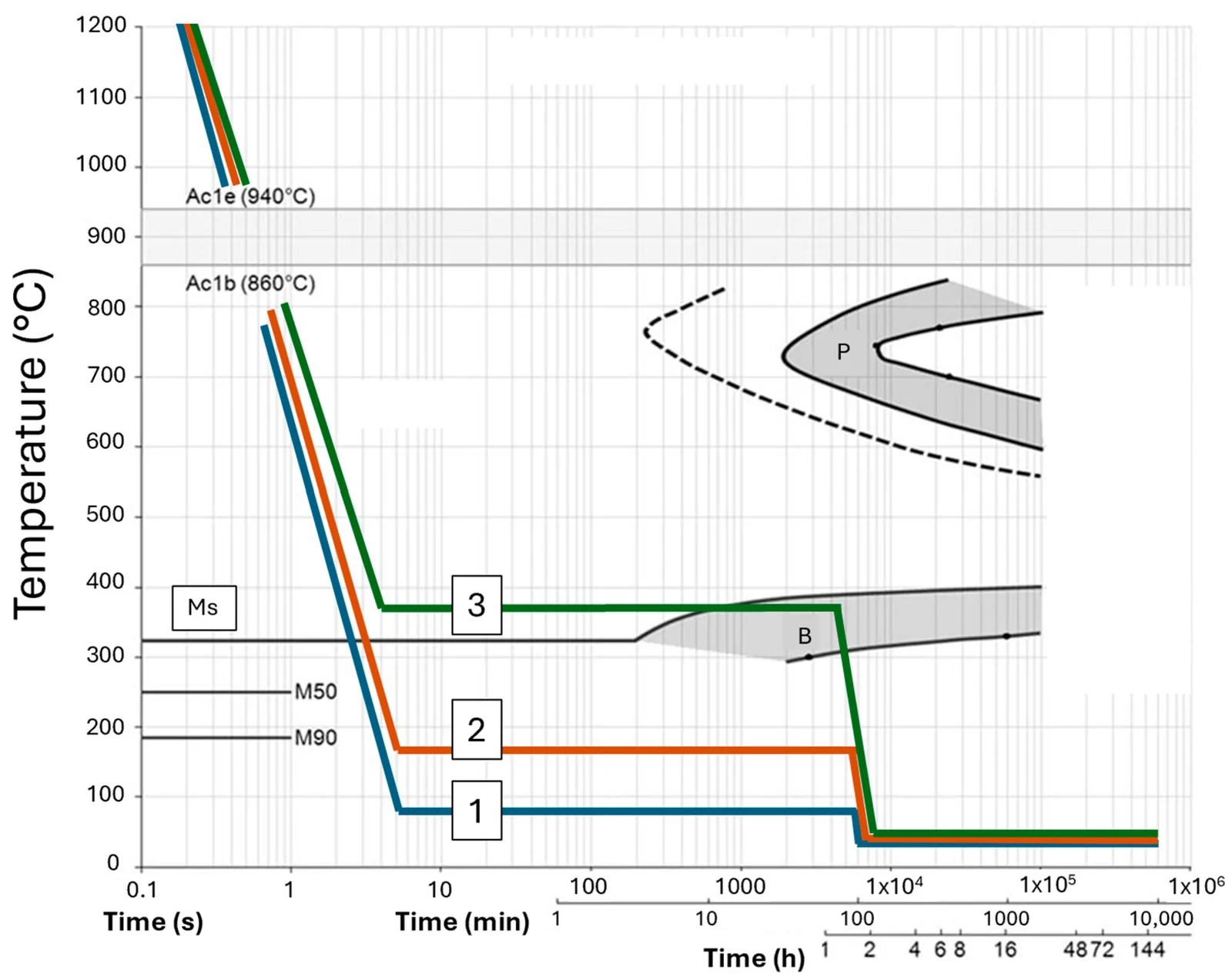
A schematic diagram of the macroscopic temperature history of the manufactured samples (based on data from [21]).

**Figure A1:**
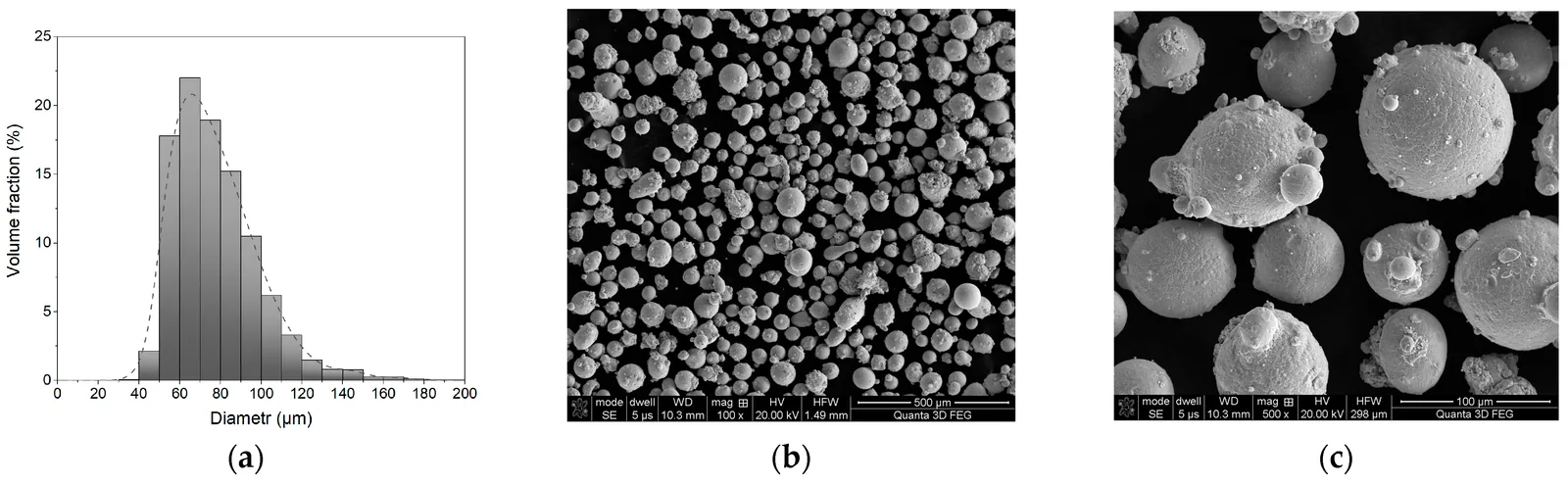
Microphotography of the powder used in the experiment: (**a**) particle size distribution of the used powder, (**b**) a lower-magnification image showing the morphology of powder particles, and (**c**) a higher-magnification image showing the morphology of powder particles.

A schematic of the macroscopic temperature change on the background of the isothermal TTT diagram is shown in Figure 2 (from the TTT diagram of H13 steel) [21]. The feedstock for building the samples was a spherical powder of X40CrMoV5-1 tool steel (TLS Technik GmbH & Co., Niedernberg, Germany) with a particle size range of 45–100 μm, as confirmed using an IPS UA particle size analyzer (KμK Instruments, Warsaw, Poland) shown in Figure A1 in Appendix A. The chemical composition of the powder measured is shown in Table 1; carbon was not measured due to inadequate EDS accuracy.

For microscopic examination, metallographic specimens were prepared using grinding and mechanical polishing. Samples for microstructure analysis were etched by swabbing with the Mi19Fe reagent, which consists of 3 g of FeCl_3_, 10 mL of HCl, and 90 mL of C_2_H_5_OH. The fabricated H13 steel samples were examined for structure porosity using an ECLYPSE NA200 (Nikon Inc., Melville, NY, USA) digital microscope at 100× and 500× magnification. Digital microscopy and a Hitachi SU8000 (Hitachi High Technologies company, Krefeld, Germany) scanning electron microscope were used to determine the microstructure of the alloys in relation to the substrate temperature. Chemical composition analysis and crystallographic orientation analysis in the micro-areas were performed using a detector equipped with an X-ray energy-dispersive spectrometer (EDS) and electron backscatter diffraction (EBSD) detectors on a Quanta 3D FEG DualBeam scanning electron microscope (FEI Quanta 3D, Hillsboro, OR, USA). X-ray diffraction (XRD) phase analysis of the fabricated alloys was performed using a Rigaku ULTIMA IV diffractometer (Ultima IV Rigaku, Tokyo, Japan), with CoKα radiation in the range of 20–140°, a voltage of 40 kV, and a current of 40 mA. A scanning rate of 1°/min and a resolution of 0.02° were used together with a fast linear detector D/teX Ultra (Rigaku, Tokyo, Japan)). The obtained results were processed using the PDXL software (Rigaku, Tokyo, Japan, version 2.8.4.0) and the ICDD PDF4+ crystallographic database (International Centre for Diffraction Data: Newtown Square, PA, USA).

**Figure A2:**
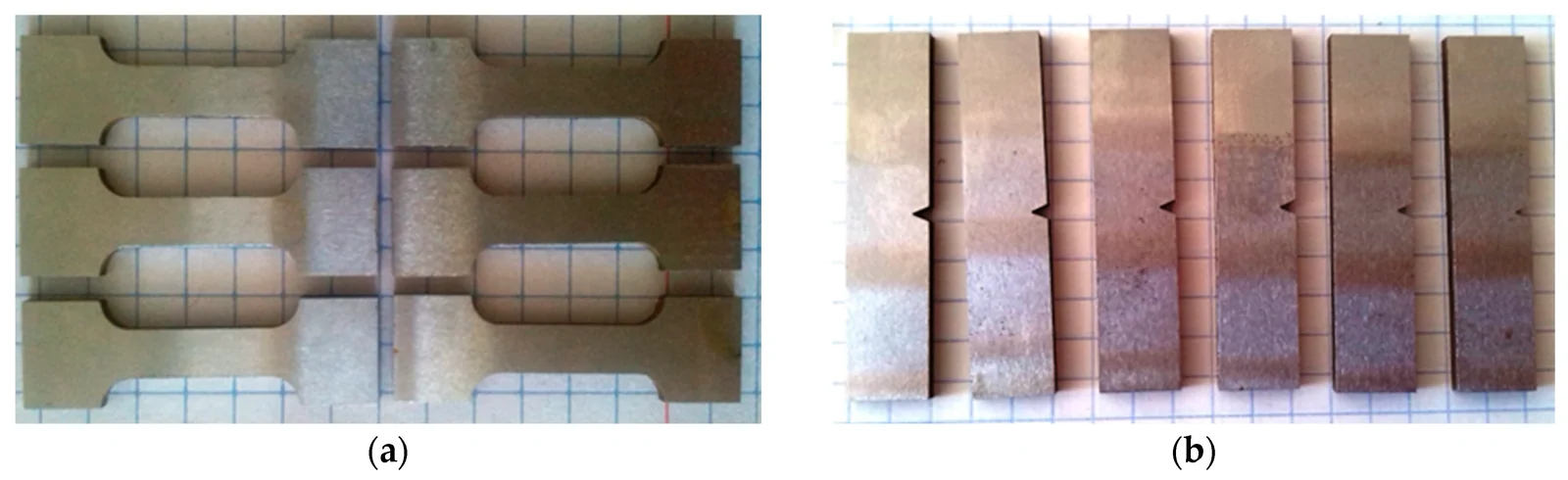
Appearance of H13 steel samples to determine their mechanical properties (a grid of 5 mm): (**a**) specimens for static tensile testing and (**b**) specimens for impact test.

The mechanical properties of the H13 alloy produced at different substrate temperatures were determined using hardness measurements, a tensile test, and an impact test. Before the mechanical properties were determined, the fabricated samples were examined for cracks and other discontinuities using a Nikon/Metris XT computer microtomograph (Nikon/METRIS XT H 225 ST, Leuven, Belgium). The appearance of H13 steel samples prepared for determining mechanical properties is shown in Figure A2 in Appendix A. Hardness was measured using the Vickers method with a Brinell–Vickers HPO-250 universal hardness tester at a load of 5 kG (49.03 N). For each specimen, measurements were taken at three specimen heights: at the base, the center, and the top. The results are presented as the mean value of 15 measurements along with the standard deviation. The Charpy impact test was performed using a Wolpert/Instron PW30 impact hammer with an impact energy of 300 J. Impact test specimens with external dimensions of 45 mm × 10 mm × 2.5 mm were cut using EDM and then ground using a surface grinder. Six measurements of impact energy (K) were performed for the specimens produced on the substrate at 80, 180, and 350 °C. Tensile parameters under uniaxial loading were measured using an Instron 8501 universal testing machine. Flat specimens with standardized geometry and external dimensions of 36 mm × 13 mm were prepared. The initial gauge length for tensile testing was 10 mm. Fracture surfaces after both the impact and static tensile tests were examined using a Quanta 3D FEG DualBeam scanning electron microscope (FEI Quanta 3D, Hillsboro, OR, USA).

## 3. Results

**Figure 3:**
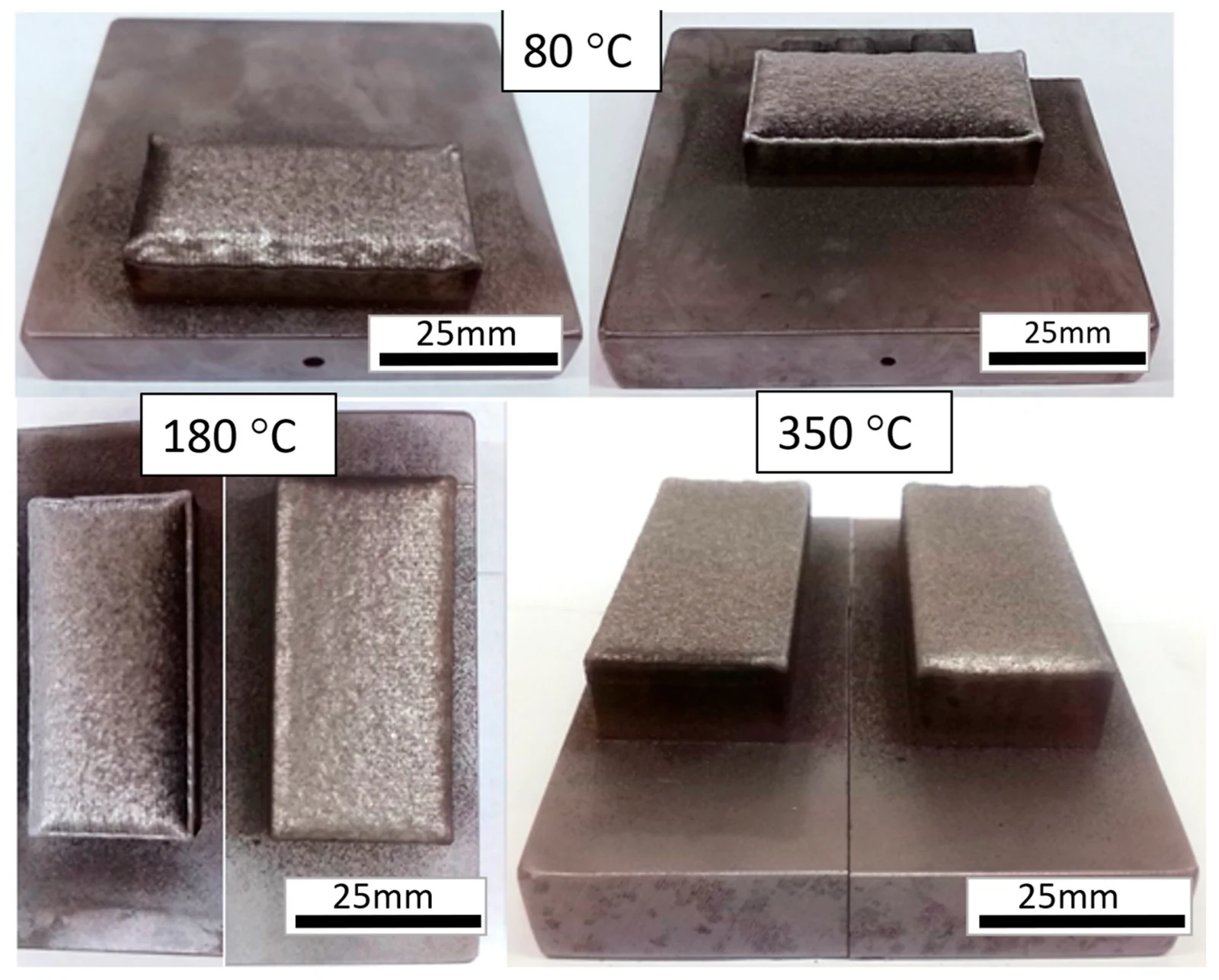
Appearance of samples produced at the initial substrate temperature and annealing temperatures of 80, 180, and 350 °C.

The appearance of H13 alloy samples produced using the LENS^®^ technique is shown in Figure 3. The porosity fraction and metallurgical quality of the fabricated H13 steel samples were determined using cross-sections of digital micrographs. No cracks or microcracks were observed, whereas porosity was determined to be ~1.4%, 1.7%, and 1.9% for samples with applied preheating and heat-treatment temperatures of 80, 180, and 350 °C, respectively. The size of the observed micropores was in the range of 0.5–1 µm. All the samples were characterized by consistent structures and full remelting of the alloy powder.

**Figure 4:**
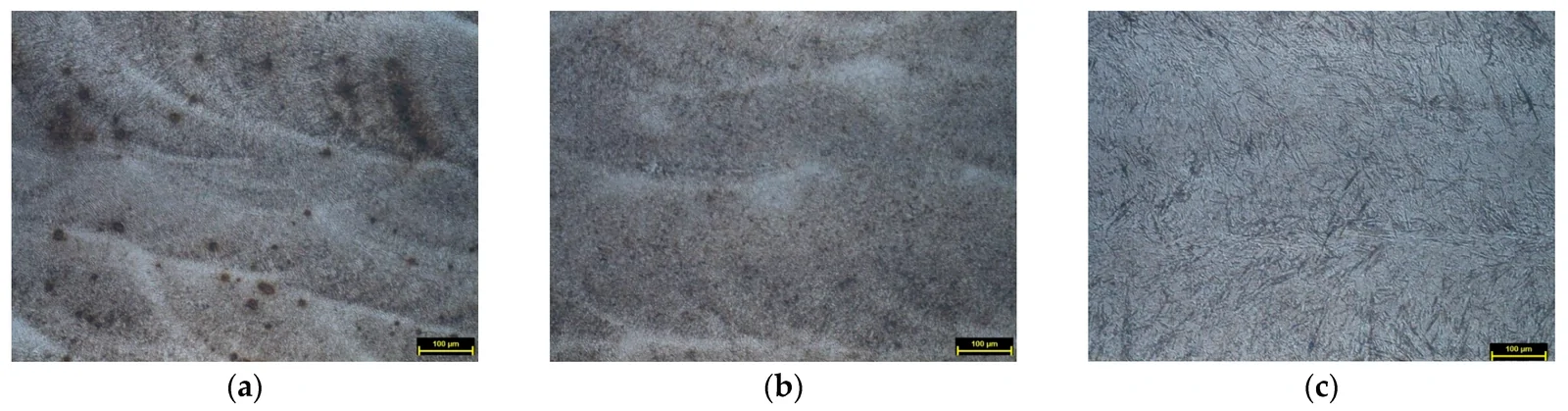
Microstructure of H13 steel with preheating and annealing temperatures of (**a**) 80 °C, (**b**) 180 °C, and (**c**) 350 °C (marker size: 100 μm).

The general microstructure of H13 steel produced at different substrate temperatures on digital micrographs at 100× magnification is shown in Figure 4. For the steel preheated and tempered at temperatures of 80 and 180 °C, the poor melt boundaries were clearly observed along the build direction and heat-affected zones (HAZs), which is typical of the microstructure of alloys produced using additive manufacture techniques [22]. For samples tempered at 350 °C, the HAZs were less noticeable but still visible on the background of the needle-like martensitic structure.

**Figure 5:**
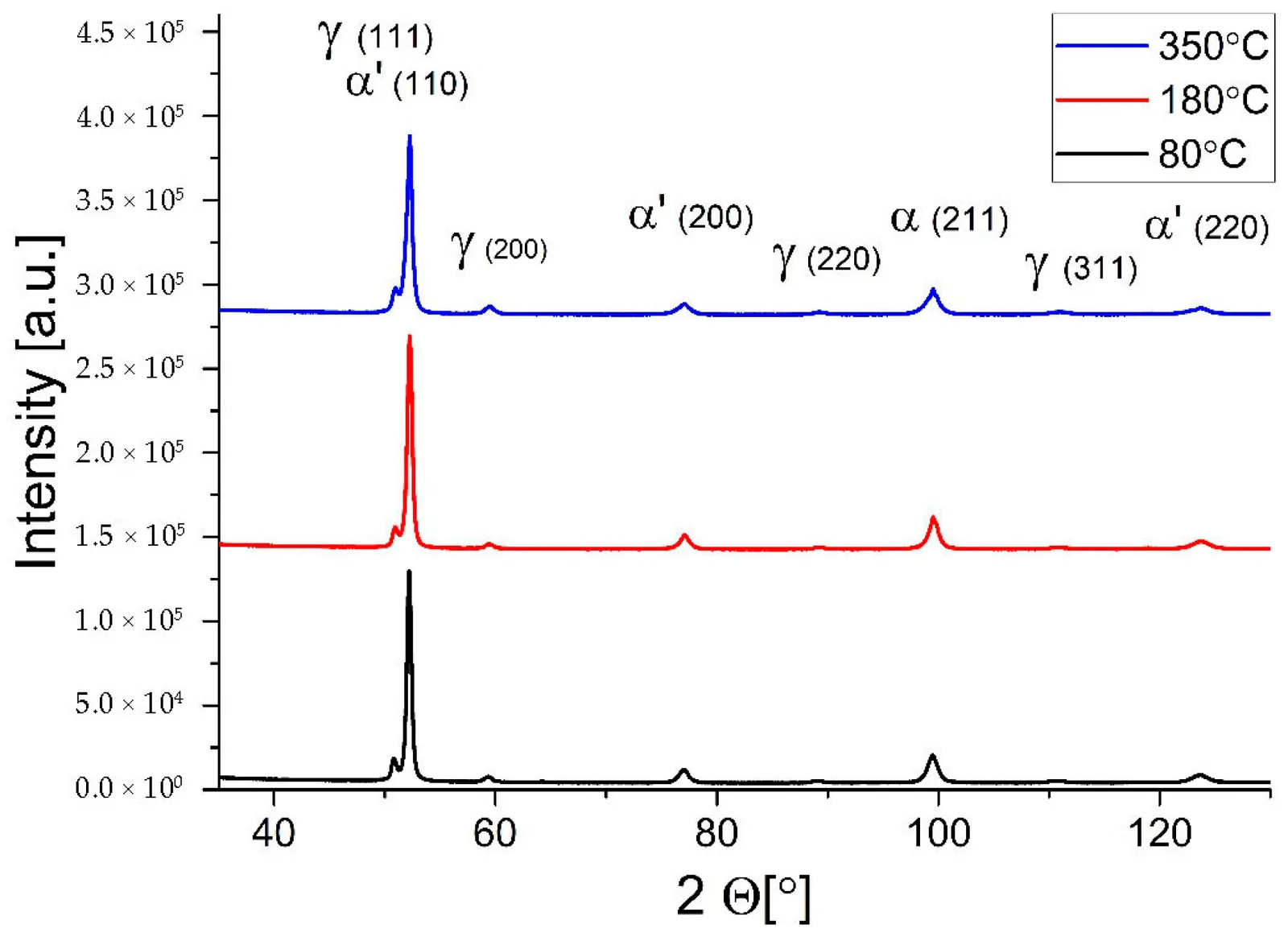
Results of the structural XRD phase analysis of H13 steel produced at substrate preheating and annealing temperatures of 80 °C, 180 °C, and 350 °C.

To identify the phases present in the fabricated samples, we performed XRD phase analysis, resulting in the diffractograms shown in Figure 5. The reflections primarily showed the presence of the α’ (martensite) phase. Considering the intensity of the peaks obtained, martensite was the dominant phase with a small addition of residual γ-austenite. Regardless of the substrate preheating and annealing temperatures, phase composition analysis did not reveal the presence of carbides in the structure of the produced H13 steel.

**Figure 6:**
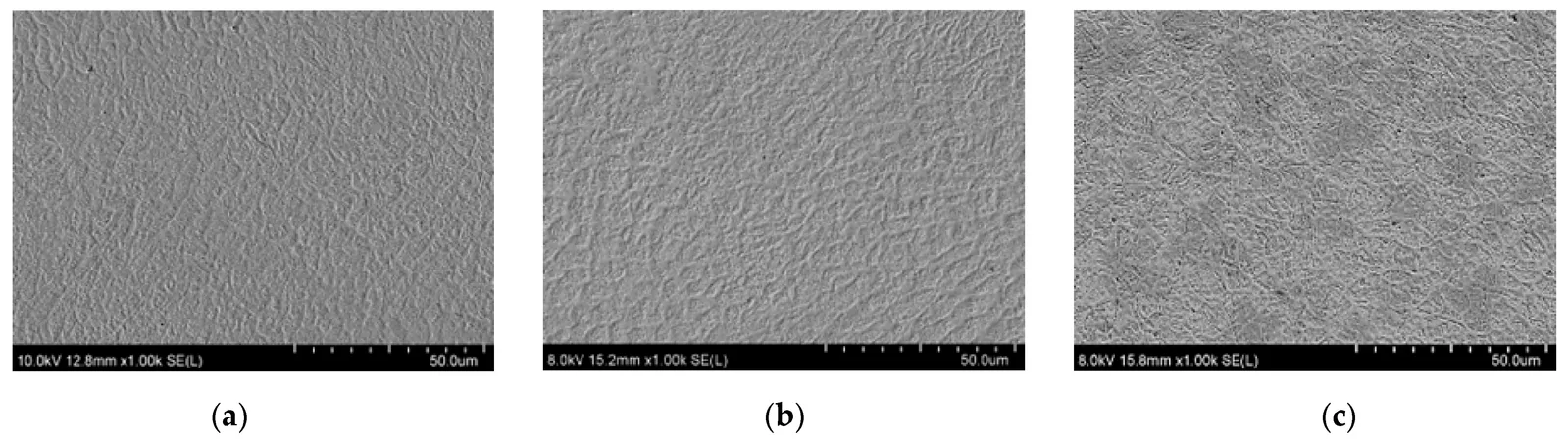
Microstructure of the H13 alloy produced at substrate preheating and annealing temperatures of (**a**) 80 °C, (**b**) 180 °C, and (**c**) 350 °C (magn. 1000×).

**Figure 7:**
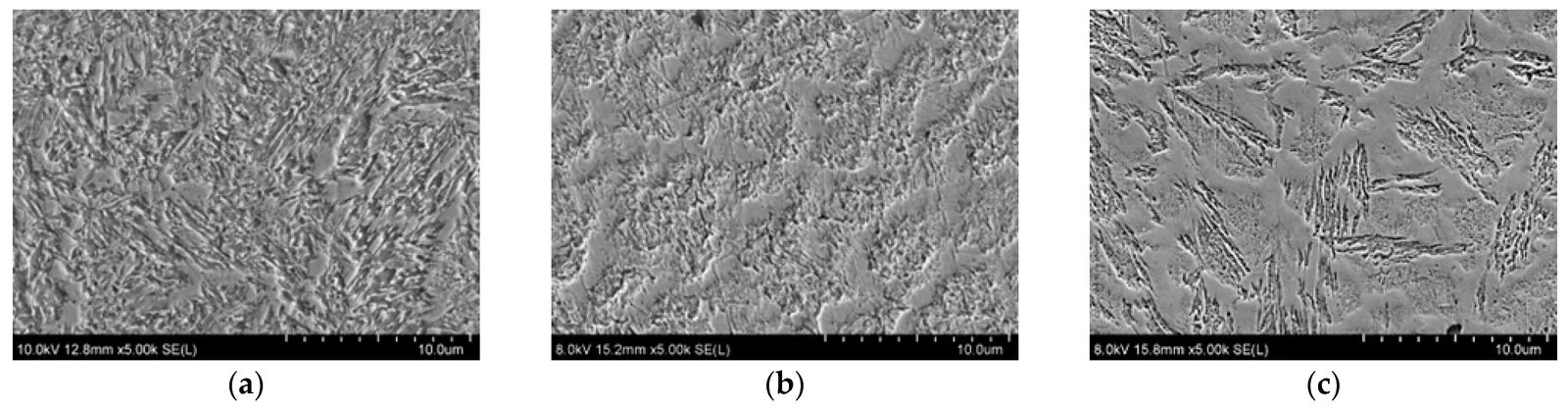
Microstructure of the H13 alloy produced at substrate preheating and annealing temperatures of (**a**) 80 °C, (**b**) 180 °C, and (**c**) 350 °C (magn. 5000×).

The microstructure of the alloy produced using LENS^®^ was fine-grained for each variant of preheating temperature used. However, some differences were visible. The microstructure of the etched alloys observed in the SE mode at 1000× and 5000× magnification is shown in Figure 6 and Figure 7. SEM micrographs of samples tempered at 80 and 180 °C showed a similar homogeneous fine-lath martensitic structure visible between the residual cellular/dendritic solidification structure, whereas the alloy tempered at 350 °C revealed a homogeneous structure composed of martensite and bainite.

**Figure 8:**
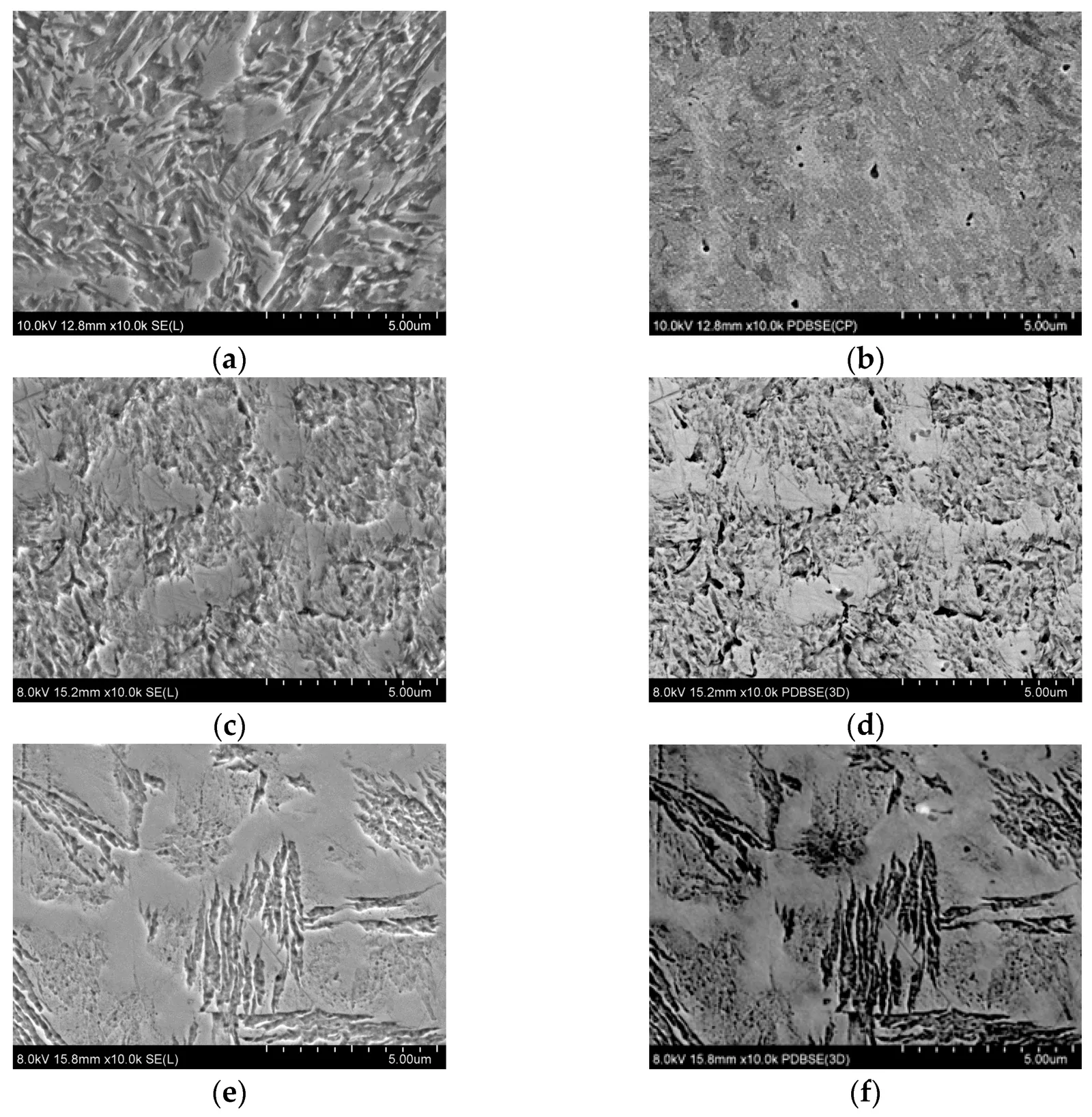
Microstructure of the H13 alloy produced at substrate preheating and annealing temperatures of (**a**,**b**) 80 °C, (**c**,**d**) 180 °C, and (**e**,**f**) 350 °C (magn. 10,000×).

Figure 8 shows micrographs of the samples captured in BSE mode and corresponding images in SE mode, showing selective dissolution of some structural components of the alloy at a magnification of 10,000×. Based on the observations using the scanning electron microscope, a tempered martensite structure with a high level of refinement was obtained for the samples preheated and maintained at 80 and 180 °C. In the BSE mode, a cellular structure was observed, which probably reflected the primary austenite grain boundaries. Martensite plates were visible inside the cells. No carbide precipitates were identified in the structure of the produced samples. At 350 °C, the structure of the H13 steel differed from that of the steels annealed at a lower temperature. In addition to martensite, typical areas of selectively dissolved bainitic structures were observed [23].

**Figure 9:**
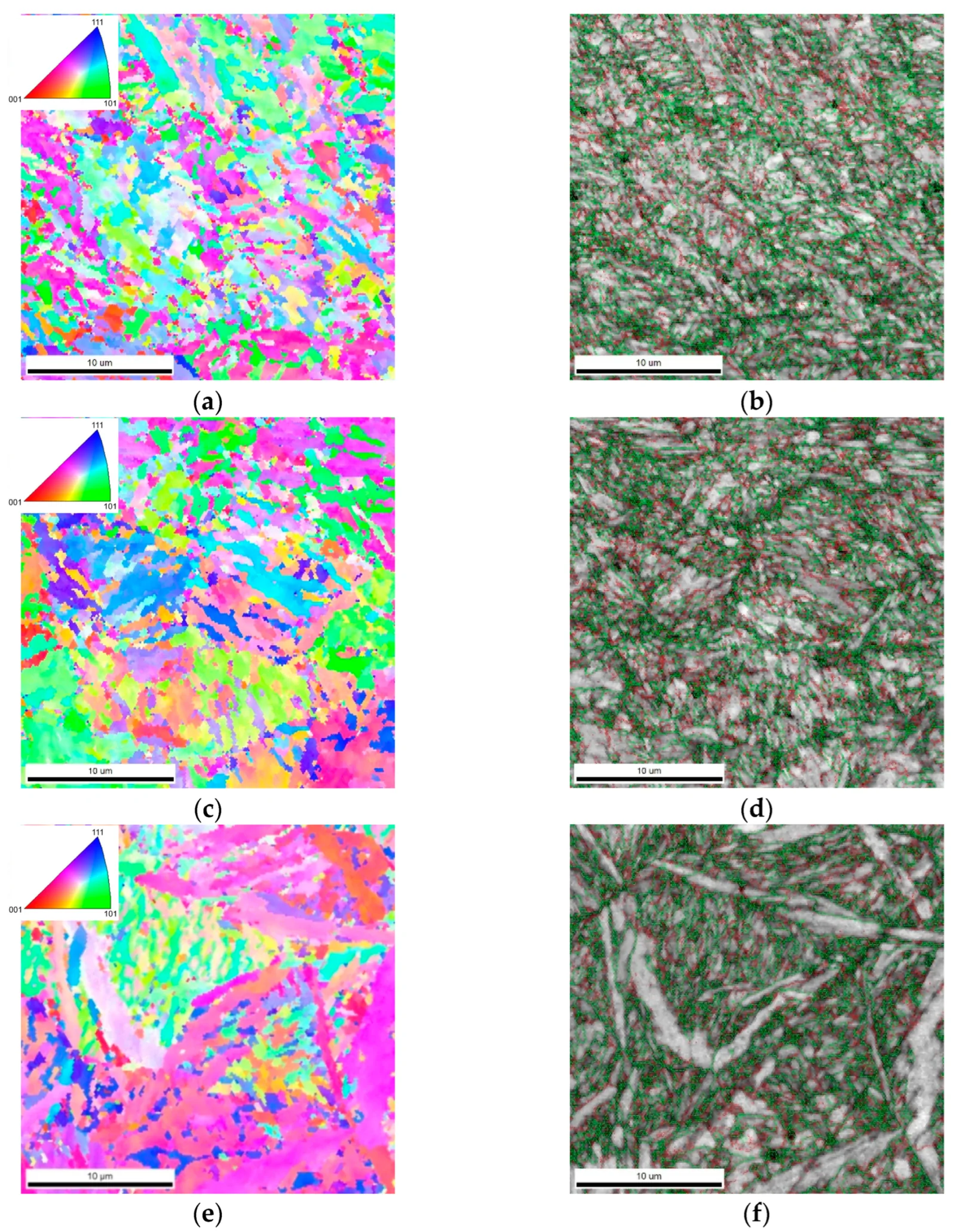
OIM maps and the corresponding IQ (image quality) maps of the samples produced at substrate preheating and annealing temperatures of (**a**,**b**) 80 °C, (**c**,**d**) 180 °C, and (**e**,**f**) 350 °C.

Figure 9 shows image quality (IQ) images compared with IPF EBSD images of the analyzed samples in their central parts. The IQ maps indicate boundaries with a misorientation angle of both up to (red) and over 20 degrees (green). Based on the results, we observed that for preheating temperatures of 80 and 180 °C, randomly oriented plates of martensite were obtained. The prior-austenite grains (primary boundaries) where the martensitic transformation occurred were also visible. The share of residual austenite in samples produced at 80 and 180 °C was determined to be 2.1%. For the alloy tempered at 350 °C, the boundaries of the primary austenite grains, in which the phase transformation occurred, were also visible. The volume of prior-austenitic grains showed both laths with a visibly different crystallographic orientation compared to the neighboring structure and packets of laths with a very similar orientation. This indicated the presence of bainite in addition to martensite. The criterion to distinguish upper from lower bainite considers the analysis of angles of misorientation (grain boundary misorientation distribution) as upper bainite exhibits a greater number of low-angle boundaries (<20°), whereas lower bainite exhibits a high proportion of boundaries with misorientations in the range of 50–60° and very few boundaries with low misorientations. EBSD IPF images are very sensitive to internal grain disorientation. For example, grains with a uniform color and no subgrain boundaries inside, compared with others that have slight color differences and subgrain boundaries inside, can be used to distinguish ferrite and bainitic ferrite, respectively [24]. The share of low-angle (<20°) boundaries in the analyzed image of the sample annealed at 350 °C was 39%. The fraction of retained austenite in the specimen tempered at 350 °C was determined to be 0.8%, but quantitative analysis using EBSD digital image analysis is subject to uncertainty owing to the deformation of the structure and the large number of boundaries in martensitic structures that may be left unresolved. No carbide precipitates were observed in the structure of the produced H13 steel samples regardless of the preheating and annealing temperatures used.

**Figure 10:**
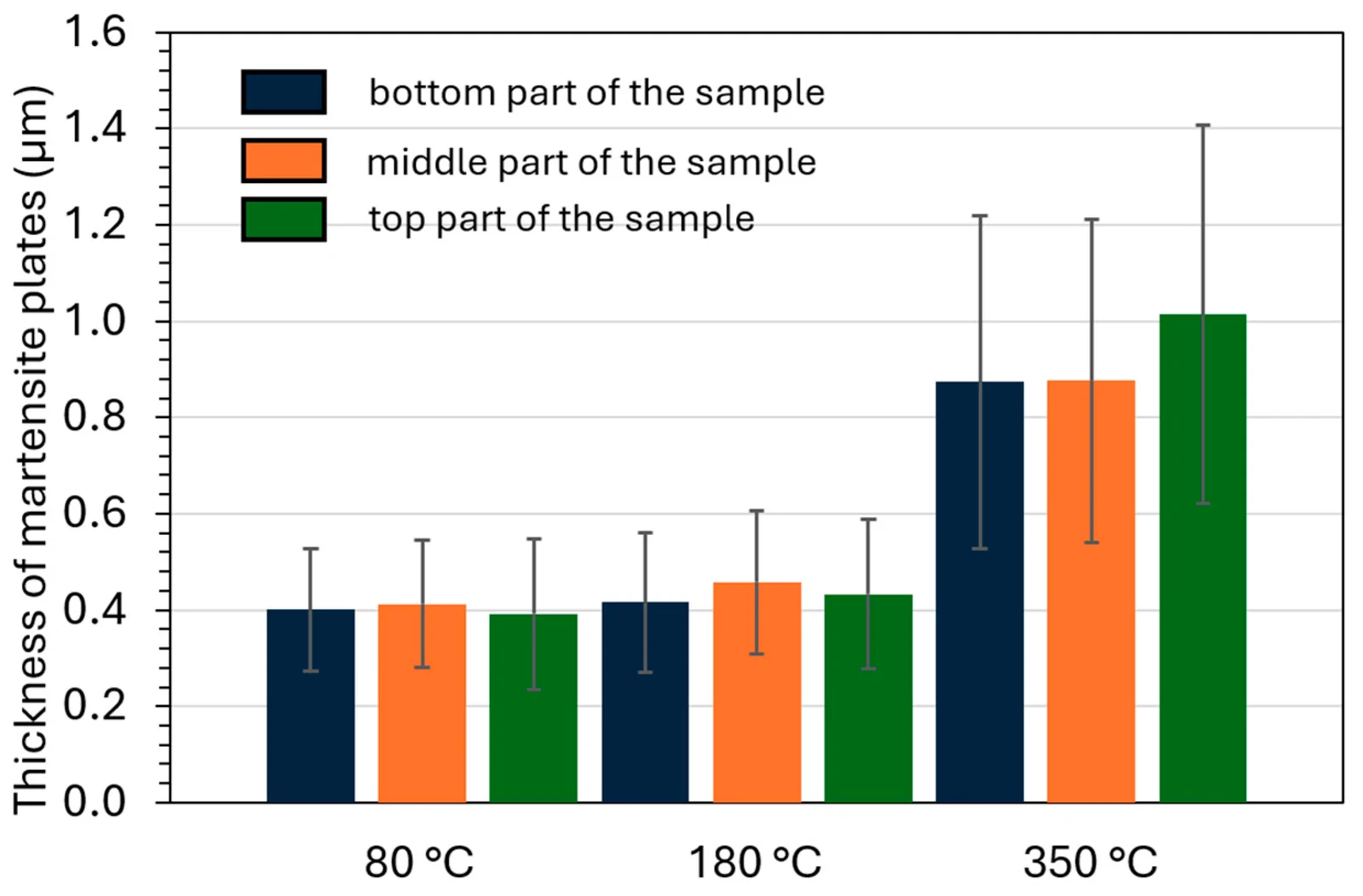
Average size of martensite plates depending on the substrate and annealing temperatures used.

Using the software TSL OIM Analysis 7, we determined the average size of martensite plates in different locations of the manufactured samples based on the distance between the high-angle boundaries: at the substrate, in the center of the sample, and at the top part of the sample. The obtained values of the average martensite plate size, together with the standard deviation, depending on the substrate and annealing temperatures used, are shown in Figure 10. At a substrate temperature of 350 °C, a larger grain size was obtained than at 80 and 180 °C. This was caused by differences in the crystallization rate of the alloy depending on the cooling rate during sample fabrication. In each manufacturing condition used, a fine-grained structure was obtained. However, the lower temperature gradient at 350 °C resulted in an average tempered martensite plate size of 0.9–1 μm, compared with 0.4 μm obtained at lower temperatures. No differences in martensite plate size were observed with respect to the height of the sample.

**Figure A3:**
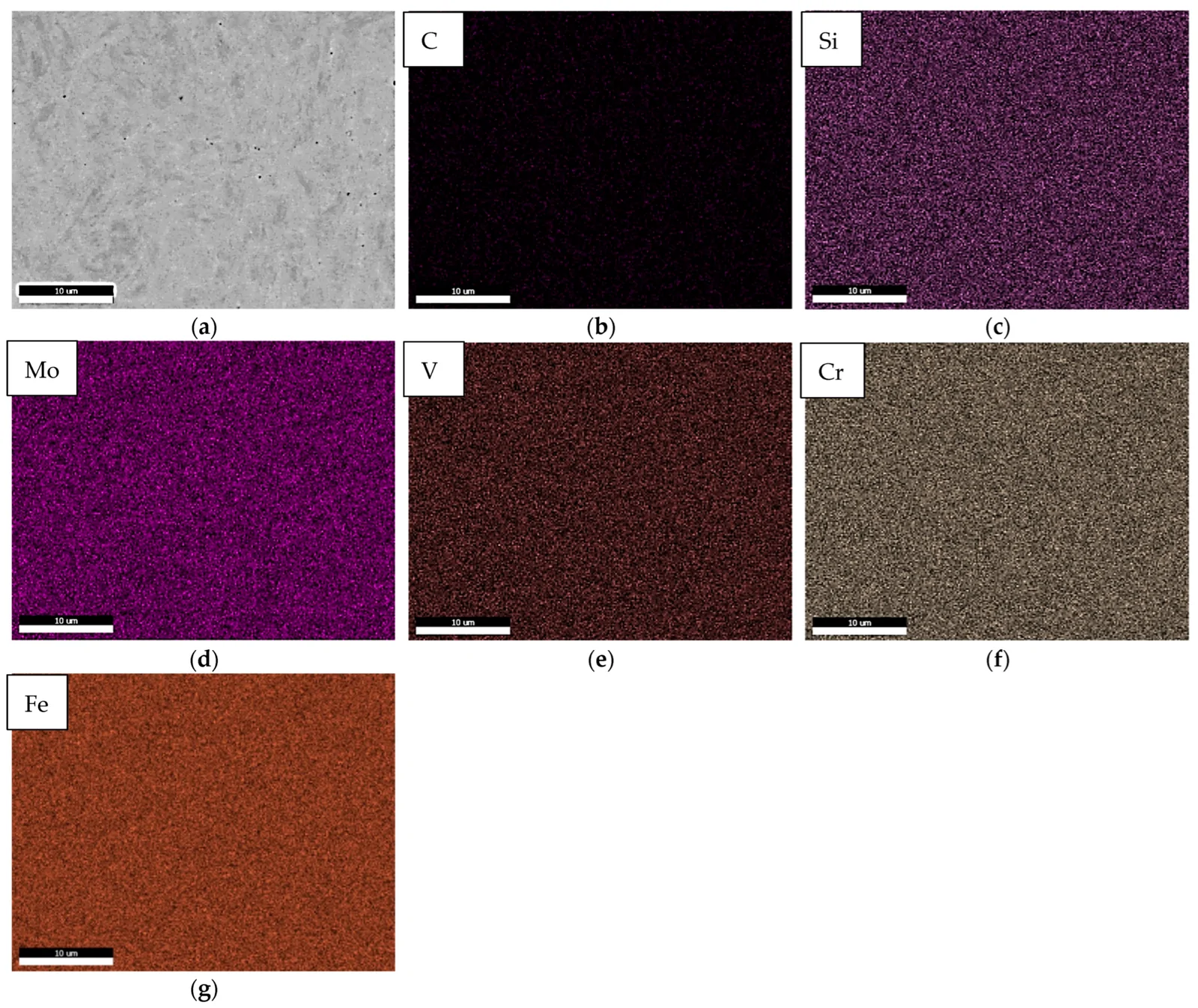
(**a**) SEM-BSE image of the sample produced at a substrate preheating and annealing temperature of 350 °C; surface distribution of individual elements: (**b**) C, (**c**) Si, (**d**) Mo, (**e**) V, (**f**) Cr, and (**g**) Fe.

No carbide precipitates were observed in EBSD images of the structure of the H13 steel samples regardless of the preheating and annealing temperatures used. The absence of carbides in the structure of the fabricated H13 alloy samples was also confirmed by conducting surface microanalysis of the chemical composition using an EDS. Chemical composition maps of the individual elements analyzed on the cross-section of the sample annealed at 350 °C are shown on Figure A3 in Appendix A. A lack of segregation of carbon and carbide-forming alloying elements, primarily V, Cr, and Mo, was observed. The same behavior was noted from all temperature variants of the substrate.

For each variant of preheating and annealing temperatures, the hardness of the produced H13 steel was measured using the Vickers method at three heights of the specimen: at the substrate, in the middle, and at the top of the specimen. The results of the measurements are shown in Table 2. Regardless of the variant of annealing temperature used, the average hardness of the alloy was slightly higher near the substrate than at the top of the sample. The measurement results also showed that changing the substrate temperature from 80 to 180 °C had no significant effect on the hardness of the H13 steel, and the average hardness values at the center of the sample were 576.4 and 574.0 HV, respectively. At an annealing temperature of 350 °C, the average hardness of the H13 steel was slightly higher than for the annealing temperature below the martensite start temperature (Ms) and reached 606.9 HV.

**Figure 11:**
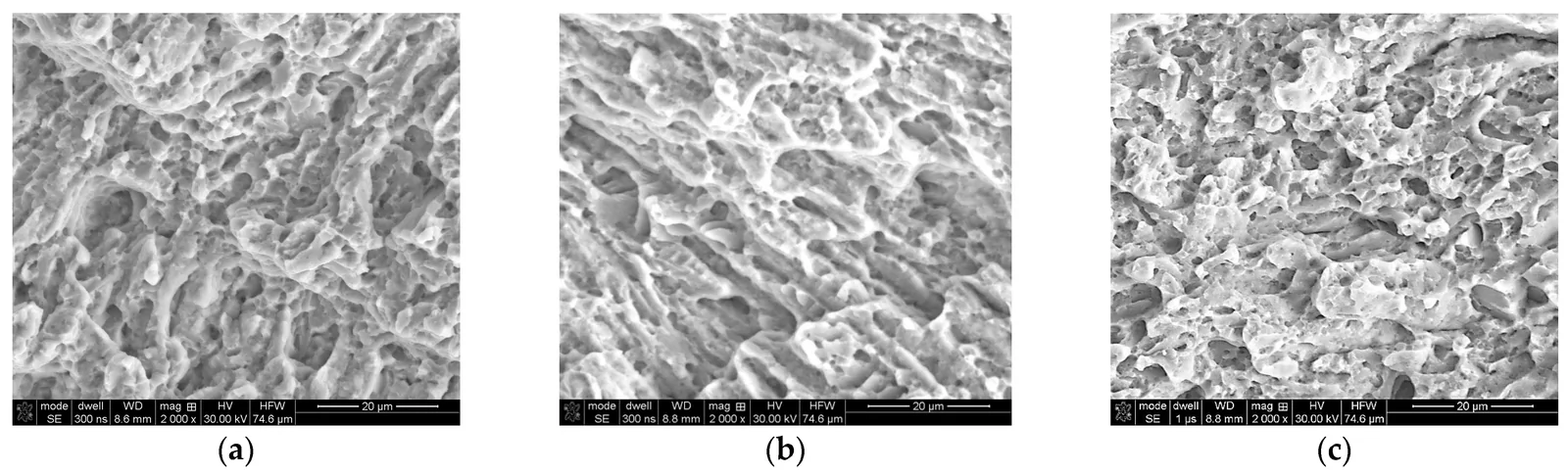
Fractures of the H13 steel annealed at (**a**) 80 °C, (**b**) 180 °C, and (**c**) 350 °C after the impact test.

The results of impact toughness measurements of the fabricated samples are shown in Table 3. The impact toughness values were 11.0 and 11.7 J/cm^2^ for samples annealed at 80 and 180 °C, respectively, and 9.8 J/cm^2^ for samples annealed at 350 °C. The obtained fractures (Figure 11) had mixed-mode transgranular quasi-cleavage characteristics with areas of localized plastic deformation. Locally, characteristic dimples were visible, and the cracks propagated intergranularly in interdendritic spaces. No carbides that could contribute to the low impact strength by increasing brittleness were observed.

**Figure 12:**
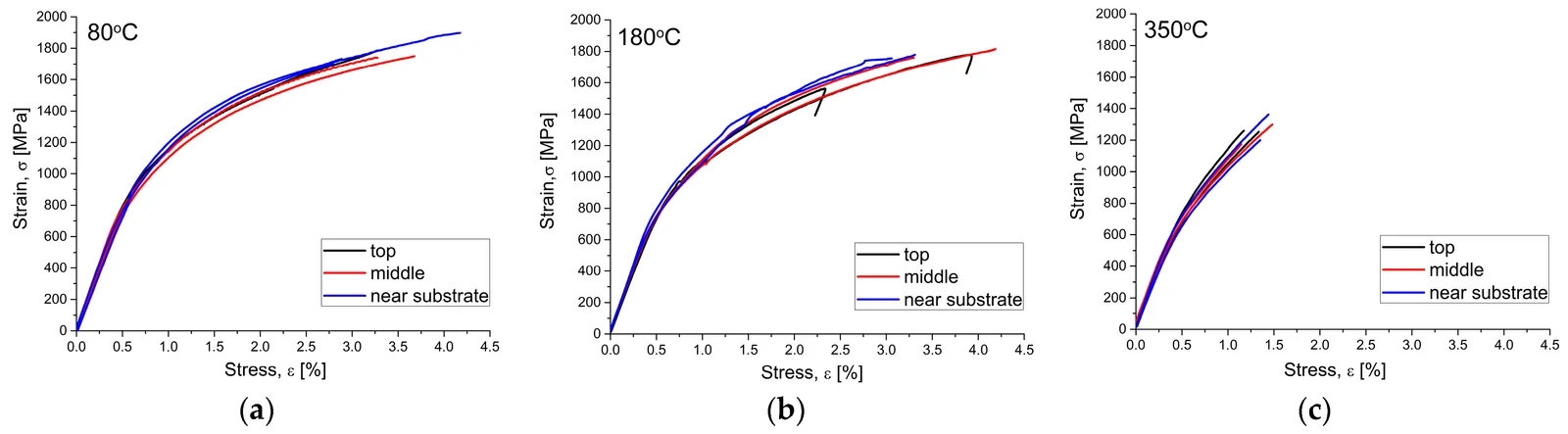
Tensile test curves of the H13 steel in situ annealed at (**a**) 80 °C, (**b**) 180 °C, and (**c**) 350 °C.

**Figure 13:**
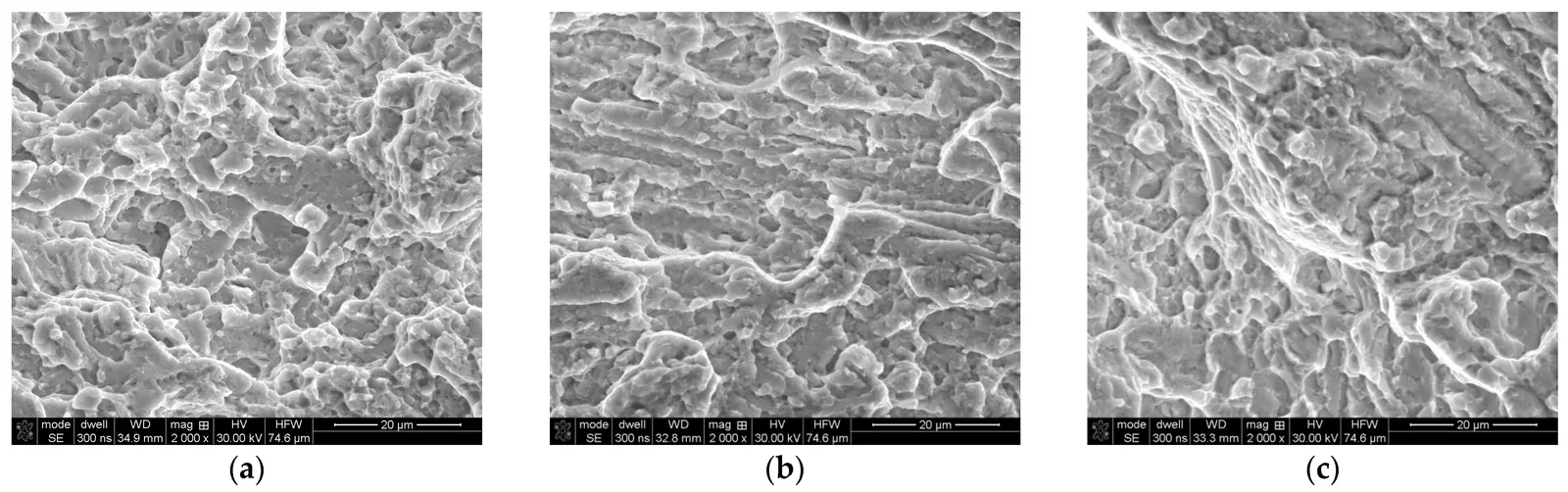
Fractures of the H13 steel annealed at (**a**) 80 °C, (**b**) 180 °C, and (**c**) 350 °C after the static tensile test.

The curves obtained during the static tensile test are shown in Figure 12. The obtained curves were characterized by the absence of a clear yield point. The values of the UTS, yield strength (YS), and elongation (A) were determined, and the results are summarized in Table 4. For specimens annealed at 80 and 180 °C, no significant differences in mechanical properties were observed. The results of UTS above 1740 MPa and YS above 1020 MPa, while maintaining an elongation of about 2%, were obtained. Deposition with in situ heat treatment of 350 °C resulted in a significant decrease in ductility and tensile strength. Here, elongation at the fracture value was 0.5%, indicating extreme brittleness of the LENS^®^-produced alloy. A UTS value of 1250 MPa and a YS value of 950 MPa were obtained. The fractures after the static tensile test were characterized as a transgranular quasi-cleavage type (Figure 13). The specimen produced at a substrate temperature of 350 °C also exhibited micropores along the cleavage plane, which were undetected using CT scanning.

## 4. Discussion

Samples of X40CrMoV5-1 tool steel were fabricated using LENS^®^ technology in three variants. During fabrication, the substrate on which the samples were built were preheated to a temperature of 80, 180, or 350 °C, and after the fabrication process, this temperature was maintained for 2 h. Samples manufactured in the form of cuboids were characterized by good quality; the outer walls of the cuboids were even, and no partially melted particles associated with the surface or surface roughness, called the staircase effect and caused by layer-by-layer manufacturing, were observed [25]. Additionally, no thermal stress-induced cracks at the substrate or clear outer traces of deposited layers were observed, such as in SLM laser-clad H13 steel by Trojan et al. [5]. No protruding edge effect was observed with a high laser energy density, ranging from 150 to 480 J/mm^3^ when fabricating H13 steel samples with SLM [12]. All of the fabricated samples were characterized by a uniform structure without cracks, full remelting of the powder particles, and microporosity at a level of about 2%. A similar density was achieved for the H13 alloy during fabrication using LPBF with a preheating temperature in the range of 200–300 °C and a volume energy density of 60–70 J/mm^3^ [6]. The highest amount of pores was found in places where the laser beam changed the scanning speed and direction, but this effect was not observed in the LENS^®^-produced samples.

In the fabricated H13 alloy, no microstructural differences were observed at different heights from the substrate, which indicated the homogenization of the structure under isothermal annealing after fabrication. In all variants of the applied heating condition, a fine-grained structure of the H13 steel was obtained. XRD phase analysis indicated that, regardless of the applied annealing temperature, a BCT phase was present in H13 alloys, as well as FCC originating from residual austenite. Carbides could not be identified using XRD, which is common when analyzing H13 steels after fabrication using additive techniques and after subsequent heat treatment. The absence of secondary carbides in the diffractograms is associated with the microstructure and fine size of carbides, for example, when they are uniformly dispersed and localized along the lath boundaries or they have nanometric dimensions after fabrication and tempering at 600 °C for 1 h [15,26]. However, secondary carbides are not always present in the structure of samples fabricated using additive techniques [27]. The absence of carbides in samples fabricated using the SLM technique and preheated during fabrication at 200 °C was also observed by Narvan et al. [12], which was attributed to the high cooling rates during the SLM process, significantly restricting the diffusion mechanisms.

The microstructure of H13 steel after fabrication using additive techniques consists primarily of martensite and residual austenite for laser beam powder bed fusion (LB-PBF) [26], laser-aided DED (L-DED) [9], SLM [28], or laser cladding [4], where fine carbides were also observed in HAZs. In the fabricated LENS^®^ samples, for in situ heat treatments at 80 and 180 °C, the structure of the H13 steel contained tempered martensite characterized by fine lamellae inside the cellular subgrain boundaries of the prior austenite structure, whereas after heat treatment at 350 °C, the microstructure consisted of lower bainite and tempered martensite. Cellular crystallization occurred owing to high temperature gradients and constitutional undercooling, which is a typical phenomenon in LENS^®^-manufactured H13 steel [29]. In all variants of the LENS^®^-produced samples of H13 steel, a small proportion of residual austenite was obtained, up to 2.1%, the presence of which was confirmed using X-ray phase analysis. The presence of retained austenite was attributed to high cooling rates during the additive manufacturing process, leading to the incompletion of phase transformation of austenite [9,30,31]. Residual austenite in cellular boundaries is a result of intercellular micro-segregation of alloying elements and chemical stabilization of austenite [14]. A similar model of this phenomenon showing the hypothesized microstructural evolution of SLM-produced X40CrMoV5-1 steel was proposed by Krell et al. [11]. In the LENS^®^-produced samples, no segregation of alloying elements was observed in the micro-areas, and the share of residual austenite was relatively small compared with, for example, H13 steel produced using SLM and after additional tempering at 700 °C, where the share of residual austenite was 4% [15].

Because of its brittleness, regardless of the temperature of preheating and annealing used, the LENS^®^-produced H13 steel had worse mechanical properties than the conventionally produced steel with an ultimate strength of up to 1590 MPa and an elongation-to-break value of up to 50% (depending on the heat treatment used) [32]. LENS^®^-produced samples using preheating temperatures of 80 and 180 °C exhibited no significant differences in mechanical properties owing to a similar microstructure and the same phase composition. The significantly lower tensile strength of the H13 steel annealed at 350 °C and the extreme brittleness of the alloy, together with the higher average hardness and lower impact strength relative to the samples annealed below Ms, may indicate that the so-called first-type tempering brittleness may have occurred. This is caused, for example, by the non-uniformity of the phase transition of the tempered martensite, e.g., at the boundaries of a grain or subgrain of the cell structure after additive manufacturing. The brittleness of H13 steel after additive SLM fabrication and annealing at temperatures of 300–600 °C may be related to secondary hardening by introducing a high number of nanoscale carbides formed and pinned at the sub-boundaries in cellular structures [15]. In addition, in the given annealing temperature range, no significant changes in dislocation density in supersaturated martensite in comparison with the as-SLM-produced sample were observed, which also affected the brittleness of the alloy. In contrast, the as-produced SLM specimen with high residual stress and poor density was characterized by an impact energy of 14.4 J and an elongation-to-brake value of above 12% [27].

Isothermal heat treatment of LENS^®^-produced H13 steel above the Ms resulted in a slight increase in the average hardness of the alloy relative to samples tempered below the Ms, where a reduction in compressive residual stresses can be observed [33]. At 350 °C during heat treatment, in addition to partial relaxation of stresses generated in the material due to rapid cooling and phase transformations, structural transformations of tempered martensite and bainite formation occur, which often results in a decrease in the hardness of the H13 steel relative to steels with a martensitic structure [34]. The increase in hardness of the H13 steel after annealing at 350 °C and the brittleness of the alloy indicate that, in addition to the bainitic transformation, nanometric-scale precipitation processes may have occurred. After impact and static tensile tests on all annealing temperatures used, the same transgranular quasi-cleavage fracture was observed, with features of low ductility with few dimples, in the volume of the cellular substructure. During fracture propagation, fine-grained martensite hinders the growth of quasi-cleavage cracks. A similar fracture character was observed in H13 steel after forging at 1150 °C, spheroidized annealing, and thermal refining by quenching in water at two tempering stages (610 °C/2 h + 580 °C/2 h) [27] and in SLM-fabricated H13 steel, both in the as-fabricated state and at different tempering conditions [14].

## 5. Conclusions

The research on the structure and mechanical properties of in situ heat-treated H13 steel during LENS^®^ additive manufacturing enabled us to formulate several conclusions:

1. The application of substrate preheating during LENS^®^ fabrication enables the reduction in thermal gradient and the production of samples of good metallurgical quality (free of cracks and with an acceptable level of porosity).
2. Changes in substrate temperature during LENS^®^ additive manufacturing have a significant impact on the microstructure, phase composition, and mechanical properties of the produced alloys.
3. Controlling the substrate temperature during laser additive manufacturing affects the structural transformations of steel. Maintaining the temperature during sample production and subsequent isothermal annealing at a level below the martensitic transformation temperature results in a fine martensitic microstructure due to rapid cooling of the alloy. The manufacturing and annealing of samples at a temperature of 350 °C result in the formation of a martensitic–bainitic structure.
4. Cooling the substrate to 80 °C during the application process results in high hardness (55–56 HRC), a tensile strength of UTS = 1746 MPa, and a yield strength of YS = 1075 MPa, with a very low elongation-at-break value of 2.1% for the H13 alloy produced. Very similar results were obtained for steel annealed at 180 °C. The mechanical properties obtained are too low to meet the requirements for hot-work tool steels.
5. Heating the substrate to 350 °C during the production of H13 steel using the LENS^®^ method results in a significant increase in the hardness of the alloy (58 HRC) and a worsening of mechanical properties, including impact strength. Extreme brittleness of the alloy is observed, with an elongation at break A = 0.5%. The observed hardening and embrittlement at 350 °C may be associated with early-stage precipitation phenomena at the nanoscale (e.g., fine carbides), consistent with the literature on H13 tempered in this range; however, direct confirmation (e.g., TEM) was not performed in this study.
6. For additive manufacturing of H13 tool steel using LENS^®^, subsequent heat treatment cannot be replaced by substrate preheating due to the absence of nanosized carbides forming in the structure, which is crucial for achieving the required mechanical properties at elevated temperatures.
7. The small size of the sample, which allowed the lack of thermal gradient in practical applications, lowers the usefulness of the method.

## Figures and Tables

**Table 1:** The chemical composition of the H13 (AISI) steel powder (in wt. %).

Result	C (Nominal)	Si Measured	V	Cr	Fe
Test 1	0.40	2.05	1.30	5.50	89.83
Test 2	0.40	2.08	1.31	5.45	90.10
Test 3	0.40	1.85	1.29	5.43	90.45
Average		1.99	1.30	5.46	90.12
Std. dev.		0.125	0.010	0.036	0.311

**Table 2:** Hardness of the LENS^®^-produced H13 steel in relation to annealing temperature and sample height.

Vickers Hardness	Preheating and Annealing Temperature		
	80 °C	180 °C	350 °C
Near the substrate	584.5 ± 32.9	602.0 ± 37.0	619.7 ± 32.7
Center	576.4 ± 35.1	574.0 ± 35.0	606.9 ± 36.1
Near the top	530.8 ± 34.0	535.3 ± 50.9	592.8 ± 35.1

**Table 3:** Impact toughness of the LENS^®^-produced H13 steel in relation to the annealing temperature.

Impact Toughness [J/cm^2^]	Preheating and Annealing Temperature		
	80 °C	180 °C	350 °C
	11.0 ± 0.7	11.7 ± 0.7	9.8 ± 0.2

**Table 4:** Strength parameters obtained from the static tensile test of the LENS^®^-produced H13 steel.

Strength Parameters During Static Tensile Test	Preheating and Annealing Temperature		
	80 °C	180 °C	350 °C
UTS [MPa]	1746.0 ± 104.7	1741.8 ± 82.6	1258.3 ± 61.6
YS [MPa]	1075.3 ± 66.7	1027.5 ± 42.4	952.7 ± 55.5
A [%]	2.1 ± 0.6	2.2 ± 0.5	0.5 ± 0.1

## Data Availability

The data obtained in this experiment are available at: https://zenodo.org/records/19551684.

## Appendix A

Appendix A presents supplementary microstructural information, including the particle size distribution and morphology of the powder feedstock (Figure A1), the appearance of H13 steel samples prepared for mechanical testing (Figure A2), and elemental distribution maps of the alloy annealed at 350 °C (Figure A3), showing a lack of carbides.
