# Correction: Rothen-Chaja et al. The Effect of In Situ Heat Treatment on the Microstructure and Mechanical Properties of H13 Tool Steel Specimens Produced by Laser-Engineered Net Shaping (LENS^®^). *Materials* 2025, *18*, 5164

**DOI:** 10.3390/ma19143022

**Published:** 2026-07-14

**Authors:** Michalina Rothen-Chaja, Izabela Kunce, Agata Radziwonko, Tomasz Płociński, Julita Dworecka-Wójcik, Marek Polański

**Affiliations:** 1 Posalux SA, 18 F. Oppliger Street, P.O. Box 6075, CH-2500 Biel, Switzerland; mrothen-chaja@posalux.com; 2 Corrosion and Chemistry Department, Road and Bridge Research Institute, 1 Instytutowa Street, 03-302 Warsaw, Poland; izabela.kunce@ibdim.edu.pl; 3 Department of Functional Materials and Hydrogen Technology, Military University of Technology, 2 Kaliskiego Street, 00-908 Warsaw, Poland; agata.radziwonko@wat.edu.pl (A.R.); julita.dworecka@wat.edu.pl (J.D.-W.); 4 Faculty of Materials Science and Engineering, Warsaw University of Technology, 141 Wołoska, 02-507 Warsaw, Poland; tomasz.plocinski@pw.edu.pl

In the original publication [1], the Data Availability Statement should be updated as follows:

## Data Availability

The data obtained in this experiment are available at: https://zenodo.org/records/19551684. The authors state that the scientific conclusions are unaffected. This correction was approved by the Academic Editor. The original publication has also been updated.
